# Weight, Lifestyle, and Health during Pregnancy and Beyond

**DOI:** 10.1155/2017/4981283

**Published:** 2017-01-19

**Authors:** Hora Soltani, Debbie Smith, Ellinor Olander

**Affiliations:** ^1^Sheffield Hallam University, Sheffield, UK; ^2^The University of Manchester, Manchester, UK; ^3^City University of London, London, UK

Healthy weight and healthy lifestyle behaviours are considered as essential prerequisites for a successful pregnancy. The importance of maternal lifestyle including nutrition and physical activity in relation to the short- and long-term birth outcomes is increasingly featured in the literature [1–3]. Recently, more attention has been given to excessive gestational weight gain and obesity as they are shown to significantly increase risks of complications during pregnancy and birth as well as elevating the risk of obesity in the offspring [4, 5].

While many western countries are mainly facing the challenge of obesity, most developing countries suffer from a dichotomy of ill health, resulting from both undernutrition and a rising trend in obesity affecting mothers and their babies [6]. There is a growing appreciation of interventions including elements of health psychology and behaviour change techniques (BCTs) in supporting health professionals to guide mothers in adapting a healthy lifestyle. These are also used to inform and motivate mothers to improve lifestyle during pregnancy to achieve healthier birth outcomes.

This special issue includes 10 articles from various parts of the world, presenting research findings related to gestational weight management and behaviour change during and after pregnancy.

Five articles focused on gestational weight management. One study highlighted the relationship between first-trimester weight gain and overall gestational weight gain (GWG) as well as showing the impact of racial differences so that Latina women gained more weight during pregnancy than their White counterparts in the United States of America. This could offer interesting insights for developing GWG interventions sensitive to the needs of women in different BMI categories and ethnic backgrounds. Another study, using a qualitative approach, explored barriers to appropriate GWG and suggested a lack of sufficient knowledge about pregnancy weight gain goals and family pressures for “eating for two” as major obstacles in maintaining a healthy weight gain during pregnancy.

The other two studies in this category assessed attitudes and experiences towards weighing during pregnancy from women's and clinicians' perspectives. The former reported that majority of women did not express any objection to being weighed during pregnancy but they indicated that there is a state of confusion and distrust with antenatal weight management interactions. The latter article suggested that while many clinicians support routine weighing during pregnancy, there are certain barriers such as inadequacy in systems and resources and lack of sufficient evidence in benefits of routine weighing which should be considered before its implementation. The last study in this category analysed the existing literature and highlighted the importance of effective BCTs in achieving healthy dietary behaviours during pregnancy. This study showed that although the reporting on the use of BCTs is very poor in the existing literature, the most commonly used BCTs in support of gestational weight management in trials with some evidence of effectiveness include “feedback and monitoring,” “shaping knowledge,” and “goals and planning.” Taken together, these five articles make important suggestions for future intervention development.

Related to the articles on gestational weight management were two articles on maternal obesity. Firstly, the association between neighbourhood poverty level at menarche and prepregnancy obesity for African American women was explored. Prepregnancy obesity was found to be higher in those women who had their first period when living in a neighbourhood where a large number of people lived below the federal poverty level. This study highlights the need to take a public health approach to maternal obesity and to look at women's lifespan and not just their pregnancy.

Secondly, maternal obesity is associated with higher risks of gestational diabetes mellitus (GDM), but how GDM is best diagnosed is currently debated. One included study examines how two different criteria may affect GDM prevalence. They audited the prevalence of Australian women with GDM using the International Association of Diabetes and Pregnancy Study Groups (IADPSG 2010) criteria compared to the Australian Diabetes in Pregnancy Society (ADIPS 1991). Results showed that 3.4–3.5% of women were diagnosed with GDM, with no difference between the two criteria.

Lastly, three articles focused on lifestyle behaviours during and after pregnancy. One study examined dietary habits in American pregnant women. Results suggested that only one in five women consume fish, potentially missing out on omega-3 which may benefit the mother and the foetus. Dietary behaviours in pregnancy and after birth were examined by ethnicity of the mother in another American study which found that fast food (associated with high saturated fat and salt intake) was eaten more frequently by Black women than White and Hispanic women in this study. These two studies together provide information for future intervention development regarding healthy eating in pregnancy.

Although there are health benefits to postnatal physical activity, women often struggle to regain fitness after birth and may not engage in physical activity. One of the included studies reports on the secondary outcome measures of a postnatal physical activity intervention designed using health psychology theory (Transtheoretical Model) and behaviour change techniques (including goal setting). No impact was found on women's body composition and well-being, and the authors report the need for further research in the area.

In summary, we believe the studies presented in this special issue make an important addition to what is known about healthy weight and lifestyle behaviours during and after pregnancy. Collectively, the findings show the need to take a public health approach and view the impact of women's weight and lifestyle through their circumstances, including sociocultural and demographic factors such as neighbourhood poverty and ethnicity. Likewise, some included articles show the need for interventions to be targeted at individual groups to enhance their implementation success and effectiveness.

*Hora Soltani* *Debbie Smith* *Ellinor Olander*

## References

[1] Barker D. J. P., Godfrey K. M., Gluckman P. D., Harding J. E., Owens J. A., Robinson J. S. (1993). Fetal nutrition and cardiovascular disease in adult life. *The Lancet*.

[2] Aviram A., Hod M., Yogev Y. (2011). Maternal obesity: implications for pregnancy outcome and long-term risks—a link to maternal nutrition. *International Journal of Gynecology & Obstetrics*.

[3] Zhang C., Ning Y. (2011). Effect of dietary and lifestyle factors on the risk of gestational diabetes: review of epidemiologic evidence. *American Journal of Clinical Nutrition*.

[4] Lewis G. (2007). The Confidential Enquiry into Maternal and Child Health (CEMACH) saving mothers' lives: reviewing maternal deaths to make motherhood safer—2003–2005. *The Seventh Report on Confidential Enquiries into Maternal Deaths in the United Kingdom*.

[5] Symonds M. E., Mendez M. A., Meltzer H. M. (2013). Early life nutritional programming of obesity: mother-child cohort studies. *Annals of Nutrition & Metabolism*.

[6] World Health Organisation http://www.who.int/mediacentre/factsheets/fs311/en/.

